# The relationship between existential well-being and mood-related psychiatric burden in Indian young adults with attachment deficits: a cross-cultural validation study

**DOI:** 10.1186/s40359-020-0388-7

**Published:** 2020-02-24

**Authors:** Michaela Hiebler-Ragger, Shanmukh V. Kamble, Elisabeth Aberer, Human Friedrich Unterrainer

**Affiliations:** 1 Department of Psychiatry and Psychotherapeutic Medicine, Medical University Graz, Auenbruggerplatz 31, A-8036 Graz, Austria; 2 Center for Integrative Addiction Research (Grüner Kreis Society), Rudolfsplatz 9, A-1010 Vienna, Austria; 3 Department of Psychology, Karnatak University, Pavate Nagar, Dharwad, Karnataka 580003 India; 4 Department of Dermatology, Medical University of Graz, Auenbruggerplatz 8, A-8036 Graz, Austria; 5 Department of Religious Studies, University of Vienna, Schenkenstraße 8-10/5th floor, A-1010 Vienna, Austria

**Keywords:** Mood-related psychiatric burden, Attachment, Spirituality, Young adults, Mental health

## Abstract

**Background:**

Attachment and spirituality are thought to have deep evolutionary roots but are always interpreted within the framework of culture, religion and personal beliefs. While insecure attachment has been observed to be positively related with psychopathology, a positive mental health effect has often been described for spirituality. To examine the cross-cultural validation of previous research focused on Austrian young adults with Western socialization, we attempt to replicate our study examining the influence spirituality has on the connection between insecure attachment and mood-related psychiatric burden with Indian young adults.

**Methods:**

We investigated Avoidant (AV) and Anxious (AX) Attachment (ECR-RD), Religious (RWB) and Existential (EWB) Well-Being (MI-RSWB), and mood-related psychiatric burden (Anxiety, Depression, Somatization; BSI-18) in 443 (31% female) Indian young adults (age range: 18–30 years) with a Hindu upbringing.

**Results:**

Compared to young adults with a Roman Catholic upbringing in a Western socialization, Indian participants did not differ in AX and EWB but scored higher in mood-related psychiatric burden (*eta*^*2*^ = .04), AV (*eta*^*2*^ = .14), as well as RWB (*eta*^*2*^ = .28; all *p* < .01). As in previous research only AX (*β* = .40) positively predicted mood-related psychiatric burden (*ΔR*^*2*^ *= .15*, all *p* < .01), while EWB was an additional negative predictor (*β* = −.11, *p* < .05).

**Conclusions:**

Our findings emphasize the universal importance of attachment and spirituality for mental health as well as the potential influence of socialization on their development.

Furthermore, they underline that Existential Well-Being – including hope for a better future, forgiveness, and the experience of sense and meaning – appears to have a compensating effect on the relation between insecure attachment and impaired mental health.

## Background

As a fundamental drive with evolutionary importance [1], the attachment system is continuously active for the duration of the lifespan [2]. It influences every human/environmental interaction [3] and thus physiological as well as psychological development [4]. Formed by positive early family environment, secure attachment creates a ‘secure base’ from which one can explore the world while simultaneously offering a ‘safe haven’ to retreat to during times of distress [5]. In contrast, an aversive family environment promotes insecure attachment styles: Inconsistent behavior in the caregiver [6] leads to anxious attachment that implies the use of hyperactivating strategies, such as demanding care [7] or worry and rumination [8]. On the other hand, neglectful behavior in the caregiver [9] leads to avoidant attachment that is defined by deactivating strategies, for instance the inability to trust others and the need for self-reliance [10]. While insecure attachment can be linked to psychopathology (e.g., affective symptoms) [11] and substance use [12, 13], secure attachment appears to provide protection against several problematic behaviors (e.g., substance abuse) and risk beliefs (e.g., underestimation of an actual risk) [14].

### Attachment across cultures

Previous studies have already shown that culture influences childhood development [15] as well as attitudes formed about the self and others [16]. For example, with regard to parenting techniques, a universal positive impact can be attributed to parental acceptance (e.g., [17]), while parental control is more likely to be seen as supportive by adolescents in collectivistic cultures but as constraining by adolescents in individualistic cultures [18]. A related influence of culture on adult attachment seems very likely [19]. From an evolutionary perspective, highly stressful factors in the early (cultural) environment – e.g. economic hardship, harsh physical conditions – are furthermore thought to steer reproductive patterns towards short-term mating strategies that imply insecure attachment patterns (e.g., [20]), while the decreased amounts of stress encountered in (cultural) environments with rich resources facilitate the long-term reproductive patterns associated with secure attachment patterns [4].

Despite these possible cultural influences on attachment patterns, an extensive cross-cultural study by Schmitt and colleagues [19] supports the universality of an internal working model of the self and others and strengthens the role of attachment as a fundamental component of human psychology [2]: Secure attachment – in line with its normative nature – appears to be the most prevalent style in almost 80% of cultural regions [19]. Furthermore, intra-cultural differences in attachment patterns generally seem to be more extensive than inter-cultural differences (e.g., [21]).

### Protective spirituality

Much like attachment, ‘spirituality is a universal experience, not a universal theology’ (p. 116) [22]. It therefore may apply to the theism in Christianity, the polytheism in Hinduism as well as to the non-theistic beliefs of Buddhism [22]. In previous studies, spirituality has not only been linked to lower levels of affective symptoms [11, 23] but has also been suggested to support the self-management of longer-term depression [24].

Due to the progressive replacement of the primary attachment figures by friends and intimate partners [25], adolescence is a turbulent time that often coincides with ‘the age of religious awakening’ [26], defined by an augmented likelihood of religious conversion or apostasy. The relationship to God or a higher power frequently meets the parameters of attachment and may consequently confer very similar positive psychological influences [27]. In line with this, an evolutionary perspective would suggest that the experience of spirituality is linked to the same neural developments underlying the need to socialize and create communities [27].

While securely attached individuals are generally relatively stable in their spiritual beliefs, insecurely attached individuals are more prone to undergo major fluctuations [28] that correspond with distressing life events [29]. However, even individuals with insecure attachment and severe mental disorders (e.g. schizophrenia) seem to be able to profit from the attachment to spiritual entities [30]. Furthermore, for its use in prevention and treatment, it may be important to consider different aspects of spirituality not only in potential participants but also in health care professionals [31].

In this study we follow on the conceptualization of spirituality as the ‘ability to experience and integrate meaning and purpose in existence through a connectedness with self, others or a power greater than oneself’ (p. 117) [32]. Therein, the quality of the attachment to a higher power can be linked to the level of spiritual well-being [33]. In line with earlier work [34], this conceptualization integrates an immanent (i.e., bio-psycho-social) and a transcendent (i.e., spiritual) field of perception [35]. The measure used to assess this Religious/Spiritual Well-Being (RSWB) includes ‘Hope Immanent’, ‘Forgiveness’ and ‘Experiences of Sense and Meaning’ as components of an immanent Existential Well-Being (EWB), while ‘Hope Transcendent’, ‘General Religiosity’ and ‘Connectedness’ are components of a transcendent Religious Well-Being (RWB) [35]. Therein, General Religiosity can be understood as a person’s faith in connection to institutions or specific religious communities and traditions, Connectedness refers to a more deinstitutionalized form of religious belief [35]. In total, the scale therefore follows the idea of a bio-psycho-socio-spiritual model of health and disease [35]. While RWB is mainly linked to a relationship with God, EWB does not refer to a specific higher power but is linked to life satisfaction as well as a confidence in the meaningfulness of life [36]. Studies have shown that Religious/Spiritual Well-Being (RSWB) is connected to lower levels of anxiety, depression, neuroticism and suicidal ideation while simultaneously being connected to more adequate coping and more sense of coherence [35].

In our previous study on young adults with a Roman Catholic upbringing in a Western socialization [11], increased amounts of EWB and RWB were related to decreased amounts of Anxious Attachment (AX) and Avoidant Attachment (AV) as well as a decreased amount of mood-related psychiatric burden. Further regression analyses indicated that only AX, but not AV, positively predicted mood-related psychiatric burden while EWB, but not RWB, might act as a potential mediator and therefore appeared to compensate, at least partly, for insecure attachment.

### The present study

In this study, we therefore aimed to test the applicability of our previous findings on the relationship between attachment, spirituality and mood-related psychiatric burden in young adults with Western socialization [11] by investigating the same variables in Indian young adults with a Hindu upbringing.

## Methods

### Sample description and procedure

The sample consisted of 541 students and post-graduates between 18 and 30 years of age at Karnatak University, Dharwad, India. Participants were recruited randomly; the number of those declining participation was not assessed. All questionnaires were completed in a physical, pencil and paper, format. As some studies indicate that religious orientations differ in their association with mood-related psychiatric burden [37, 38], we focused on the most common religious orientation in India and therefore included only participants with a Hindu upbringing. Consequently, 56 participants with other religious orientations were excluded. After also excluding participants with missing data (*n* = 42), 443 participants with completed questionnaires and were selected for data analyses. Regarding the missing data, there was no clear pattern indicating that participants had difficulties with specific questionnaires or items.

The study was carried out in accordance with the Declaration of Helsinki. Ethical approval was granted by the Ethics Committee of Karnatak University, Dharwad, India.

### Psychometric assessment

In replication of the previous study [11], were a more extensive description of the psychometric assessment can be found, the following standardized self-report questionnaires were applied: The *Experience in Close Relationships - Revised* (ECR-RD) [39] measures ‘Avoidant Attachment’ (AV) and ‘Anxious Attachment’ (AX) with 36 items rated on a 7-point Likert scale. The *Multidimensional Inventory for Religious/Spiritual Well-bein*g (MI-RSWB) [40] measures Existential Well-Being (EWB), Religious Well-Being (RWB) and a total scale of Religious/Spiritual Well-Being (RSWB). The 48 items are rated on a 6-point Likert scale. To date, the MI-RSWB has already been extensively applied in studies of different clinical as well as healthy populations [35]. The *Brief Symptom Inventory-18* (BSI-18) [41] measures the mood-related psychiatric burden (Anxiety, Somatization and Depression) for the preceding 7 days with 18 items rated on a 5-point Likert scale. Overall, the Global Severity Index (GSI) can be generated.

### Statistical analyses

ANOVAs and ANCOVAs were conducted to examine possible differences between this sample and young adults from a Western socialization [11]. Pearson’s correlation analysis was used to examine the connection between study variables. Furthermore, hierarchical regression analyses were used to examine the influence of spirituality and attachment on all dimensions of mood-related psychiatric burden. Due to the explorative nature of the study alpha was set to *p* < .05.

## Results

### Participants

The mean age of the participants was 22 years (*SD* = 1.41). 137 (31%) were female. 75 (17%) were in a romantic relationship and 5 (1%) had children. The nationality of all participants was Indian. For highest completed education level, 3 (1%) had a high school diploma while 440 (99%) had a university degree.

### Cultural differences

To investigate possible cultural differences, we compared the present sample with a sample of young adults with a Roman Catholic upbringing in a Western socialization (92% Austrian nationality, Age: *M* = 23, *SD* = 2.93, 76% female) from our previous study [11] (see Table 1): As the samples differed in age and sex, with Indian participants being slightly younger (*F*_(1,922)_ = 30.66, *p* < .01, *eta*^*2*^ = .03) and predominantly male (*Chi*^*2*^ = 175.76, *p* < .01), we controlled for these variables by defining them as covariates in the following group comparisons. Consequently, Indian participants showed increased amounts of mood-related psychiatric burden (GSI: *F*_(1,920)_ = 35.10, *p* < .01, *eta*^*2*^ = .04) and AV (*F*_(1, 920)_ = 145.57, *p* < .01, *eta*^*2*^ = .14) but also increased amounts of RWB (*F*_(1, 920)_ = 352.59, *p* < .01, *eta*^*2*^ = .28). Interestingly, no differences in AX (*F*_(1, 920)_ = 5.52, *p* > .01, *eta*^*2*^ = .01) and EWB (*F*_(1, 920)_ = 3.14, *p* > .01, *eta*^*2*^ = .00) were found between Indian young adults and young adults with a Roman Catholic upbringing in a Western socialization.

**Table 1:** Differences in attachment, spirituality and mood-related psychiatric burden between young adults with a Hindu compared to a Roman Catholic upbringing

Measures	Hindu		Roman Catholic			
	*M*	*SD*	*M*	*SD*	*F*_*(1,920)*_	*eta*^*2*^
BSI-18						
1. Anxiety	6.69	5.20	4.57	3.94	45.69**	.05
2. Depression	6.17	5.03	5.09	5.02	9.63**	.01
3. Somatization	4.17	4.12	3.15	3.53	24.79**	.03
4. GSI	17.04	12.05	12.80	10.11	35.10**	.04
ECR-RD						
5. AX	3.19	1.00	3.11	1.20	5.52	.01
6. AV	3.39	.90	2.45	1.05	145.57**	.14
MI-RSWB						
7. EWB	108.83	12.78	107.02	15.48	3.14	.00
8. RWB	98.38	11.04	76.65	18.91	352.59**	.28
9. RSWB	203.66	20.26	183.67	29.99	109.56**	.11

**p < .01, Roman Catholic = Sample of our previous study [11], *BSI-18* Brief Symptom Inventory-18, *GSI* Global Severity Index, *ECR-RD* Experience in Close Relationships – Revised, *AX* Anxious Attachment, *AV* Avoidant Attachment, *MI-RSWB* Multidimensional Inventory of Religious/Spiritual Well-Being, *EWB* Existential Well-Being, *RWB* Religious Well-Being, *RSWB* Religious/Spiritual Well-Being

### Regression analyses

A correlation analysis (see Table 2), as well as hierarchical regression analyses (see Table 3), were conducted to explore the impact of attachment and spirituality parameters on mood-related psychiatric burden in Indian young adults.

**Table 2:** Descriptive characteristics and correlations of study variables

Measures	BSI-18				ECR-RD		MI-RSWB		
	1. Anxiety	2. Depression	3. Anxiety	4. GSI	5. AX	6. AV	7. EWB	8. RWB	9. RSWB
BSI-18									
1. Anxiety	–								
2. Depression	.62**	–							
3. Somatization	.57**	.45**	–						
4. GSI	.89**	.84**	.78**	–					
ECR-RD									
5. AX	.28**	.40**	.27**	.38**	–				
6. AV	−.01	.01	.04	−.01	.01	–			
MI-RSWB									
7. EWB	−.12*	−.19**	−.09	−.16**	−.22**	−.25**	–		
8. RWB	−.02	−.12*	−.06	−.08	−.18**	−.21**	.49**	–	
9. RSWB	−.09	−.19**	−.09*	−.15**	−.24**	−.27**	.88**	.84**	–

* *p* < .05, **p < .01, *BSI-18* Brief Symptom Inventory-18, *GSI* Global Severity Index, *ECR-RD* Experience in Close Relationships – Revised, *AX* Anxious Attachment, *AV* Avoidant Attachment, *MI-RSWB* Multidimensional Inventory of Religious/Spiritual Well-Being, *EWB* Existential Well-Being, *RWB* Religious Well-Being, *RSWB* Religious/Spiritual Well-Being

**Table 3:** Hierarchical regression analyses predicting mood-related psychiatric burden

Step and predictor variable		Anxiety			Depression			Somatization			GSI		
		*R*^*2*^	*ΔR*^*2*^	*β*	*R*^*2*^	*ΔR*^*2*^	*β*	*R*^*2*^	*ΔR*^*2*^	*β*	*R*^*2*^	*ΔR*^*2*^	*β*
Step 1		.00	.00		.00	.00		.00	.00		.00	.00	
	Sex			.04			.01			−.01			.02
Step 2		.09**	.09**		.16**	.16**		.08**	.08**		.15**	.15**	
	Sex			.09			.09			.04			.09
	AX			.29**			.41**			.28**			.40**
	AV			−.05			.01			.04			.00
Step 3		.10**	.01		.18**	.02*		.08**	.00		.16**	.01	
	Sex			.10			.10			.05			.10
	AX			.28**			.39**			.28**			.39**
	AV			−.06			−.02			.03			−.02
	EWB			−.12*			−.12*			−.03			−.11*
	RWB			.07			−.01			.01			.03

* *p* < .05, **p < .01, Dependent variables: Anxiety, Depression, Somatization, GSI; Independent variables: Sex, AX, AV, EWB, RWB; *ΔR*^*2*^ ***=*** Differences between two subsequent *R*^*2*^, *GSI* Global Severity Index (BSI-18), *AX* Anxious Attachment (ECR-RD), *AV* Avoidant Attachment (ECR-RD), *EWB* Existential Well-Being (MI-RSWB), *RWB* Religious Well-Being (MI-RSWB)

As in our previous study [11], correlation analyses showed that increased amounts of AX were related to an increased amount of mood-related psychiatric burden (*r* = .38, *p* < .01) and a decreased amount of RSWB (*r* = −.24, *p* < .01). However, while AV was also related to a decreased amount of RSWB (*r* = −.27, *p* < .01) it was not related to mood-related psychiatric burden.

In the hierarchical regression analyses, sex was entered as a control variable at Step 1 to ensure comparability with the analyses performed in our previous study [11]. AX and AV were entered at Step 2, RWB and EWB at Step 3. Including all predictors and the control variable, the hierarchical regression analyses accounted for 10% of the variance in Anxiety (*F*_(5, 441)_ = 9.47, *p* < .01), 18% of the variance in Depression (*F*_(5, 441)_ = 18.81, *p* < .01), 8% of the variance in Somatization (*F*_(5, 441)_ = 7.37, *p* < .01) and 16% of the variance in GSI (*F*_(5, 441)_ = 16.64, *p* < .01).

At every stage sex was unrelated to any dimension of mood-related psychiatric burden. At Step 2, AV did not predict mood-related psychiatric burden but AX positively predicted all three dimensions of mood-related psychiatric burden (Anxiety: *β* = .29; Depression: *β* = .41; Somatization: *β* = .28; GSI: *β* = .40, all *p* < .01). At Step 3, AV also did not predict mood-related psychiatric burden while AX still positively predicted all dimensions of mood-related psychiatric burden with *β* only slightly smaller than at Step 2. In addition, EWB was a negative predictor for Anxiety (*β* = −.12, *p* < .05), Depression (*β* = −.12, *p* < .05) and GSI (*β* = −.11, *p* < .05) but not Somatization. RWB was unrelated to all dimensions of mood-related psychiatric burden. Interestingly, only the model for Depression showed a small increase in R^2^ between Step 2 and Step 3 (*ΔR*^*2*^ = .02, *p* < .05).

## Discussion

As with our earlier research [11] that was focused on young adults with a Roman Catholic upbringing in a Western socialization, the results of this study also show a close relationship between attachment, spirituality and mood-related psychiatric burden. Specifically, an increased amount of Religious/Spiritual Well-being (RSWB) was related to less anxious attachment (AX) and less avoidant attachment (AV) while an increased amount of mood-related psychiatric burden was related to more AX as well as a decreased RSWB. Furthermore, Existential Well-Being (EWB) served as an additional negative predictor of mood-related psychiatric burden independent of AX which supports our hypothesis that spirituality influences the relationship between insecure attachment and mood-related psychiatric burden. This suggests that EWB can have a corrective effect on insecure attachment and can therefore be highly valuable to mental health. In addition, while individuals with insecure attachment generally find it difficult to rely on a higher power, the mechanisms applied by those who manage to overcome these difficulties likely also contribute to an increased well-being. As in our previous study [11], avoidant attachment (AV) as well as Religious Well-Being (RWB) seem of less importance regarding mood-related psychiatric burden than AX and EWB.

### Attachment related coping strategies

The different relation of AX and AV to mood-related psychiatric burden could be attributed to the contrasting coping strategies connected to these attachment dimensions: Dysfunctional emotional regulation [42] and heightened distress resulting from hyperactivating strategies [25] likely underlie the connection between AX and mood-related psychiatric burden. Conversely, the deactivating strategies associated with AV that are defined by a lack of visible negative emotions, as well as a high level of unconscious distress [43], might explain the missing connection between AV and mood-related psychiatric burden. The ‘segregated’ mental systems [44] associated with deactivating strategies likely lead to an exclusion of upsetting cognitions, like personal deficiencies, from consciousness. However, as deactivating strategies tend to be unreliable [45], one might argue that AV does increase mood-related psychiatric burden but that the relating symptoms are unlikely to be acknowledged by avoidant individuals. In line with this, hyperactivating strategies appear to mediate the relationship between AX and mood-related psychiatric burden while evidence for deactivating strategies in this regard is mixed [9] and will have to be explored in more detail in future studies.

### Correspondence and compensation pathway

As stated above, the relationship to a higher power can fulfil the criteria of an attachment bond and can consequently be assumed to have similar psychological advantages [27]. This association between attachment and spirituality can be explained by two hypotheses [27]: On the one hand, Bowlby’s [46] correspondence hypothesis states that mental models can generalize across various attachment relationships and therefore may also extend to the relationships with a higher power. On the other hand, Ainsworth’s [47] compensation hypothesis suggests that an attachment to a higher power may be developed as a surrogate for secure human attachment figures. Therefore, correspondence and compensation hypotheses can be seen as two pathways to as well as two ways of being religious [48]. In addition, the compensation pathway implies that secure attachment can be learned even later in life and without support from another human individual [48].

The results of this study furthermore support our previous finding [11] that only EWB, but not RWB, is related to mood-related psychiatric burden independent of attachment. The small correlation between RWB and Depression might simply occur due to the parallels in criteria and therefore psychological advantages between attachment relationships and the relationship between believers and God [27]. While the relation between lower RSWB and more insecure attachment (low AX / low AV) supports these similarities (i.e., correspondence pathway), EWB appears to offer some protection against mood-related psychiatric burden independent of attachment security. Importantly, as we only found a small independent effect of EWB on mood-related psychiatric burden, future research will have to further explore whether and under what conditions EWB can influence mood-related psychiatric burden independent of attachment security. In general, this independent effect of EWB tentatively supports the idea that the relationship to a higher power might compensate for an insecure attachment to other people (i.e., compensation pathway). In line with this, EWB – including the experience of sense and meaning, forgiveness as well as immanent hope – may be able to develop based on a previously (through secure attachment to primary caregivers and/or a higher power) established ‘secure base’ [5]. More specifically, this ‘secure base’ enables the individual to perceive the different identities of self and others so that autonomy as well as connectedness can be developed [1]. Conversely, RWB – including general religiosity, connectedness and transcendent hope – may be more closely related to social contexts and therefore to the correspondence pathway [49]. This consequently may explain why RWB did not contribute to the prediction of the mood-related psychiatric burden independent of attachment parameters.

### Methodological considerations

As this study centred on young Indian adults with a Hindu upbringing, the generalizability of our findings to young adults with different religious upbringings could be limited. Furthermore, it is beyond the scope of this study to separate the possible interconnections among culture, religion and socialization. However, the similarity in results between this study and our previous research on young adults with a Roman Catholic upbringing in a Western socialization [11] suggests a fundamental and universal connection between attachment, spirituality and mood-related psychiatric burden. In line with this, a recent study by McClintock and colleagues [50] supports a universal connection of love and interconnectedness with less risk of psychopathology. However, while spirituality was connected to less risk of psychopathology in India and the United States, it was connected to a higher risk in China [50]. With some recent models of happiness and well-being still being based on ancient (Indian) texts (e.g., [51]), future studies might also benefit from considering possible cross-cultural differences and similarities in these models.

Furthermore, the use of English questionnaires (due to the lack of appropriate translations) in the present study might have influenced the results. A replication of this study with translated questionnaires should therefore be one possible aim for future research. However, a strength of this study lies in the extensive assessment of religious and spiritual well-being, since belonging to a religious denomination is not thought to be equivalent to the engaging in spiritual and/or religious beliefs and practices [52]. Regarding attachment, our assessment of the current attachment style – although largely based on past experiences [53] – might not be as strong a predictor of spirituality. Furthermore, the inherent problem of self-report measures (e.g., defensive responding) [49] may have influenced the assessment, especially of attachment and RSWB. In addition, non-ignorable non-response (e.g., affective symptoms decreasing the likelihood of participation) as well as within-person variability in affective symptoms over time could have led to an un-representative high amount of healthy participants. Therefore, additional studies in clinical settings are needed.

Regarding the statistical analyses, alpha was set to *p* < .05 due to the explorative nature of the study. While the results have therefore to be interpreted with some caution, a more stringent alpha or different (e.g., multivariate) analyses were not applied as this may have promoted a premature exclusion of potentially relevant variables in future studies.

Lastly, studies with a longitudinal design are needed to explore all possible interactions between attachment, spirituality and mood-related psychiatric burden. For example, as insecurely attached individuals are more prone to undergo major fluctuations [28] in their spiritual beliefs that correspond with distressing life events [29], mood-related psychiatric burden might cause and/or result from lower RSWB. Interestingly, research shows that older adults with high levels of religiosity and spirituality are also less likely to have high levels of depressive symptoms [23].

## Conclusion

This research contributes to clinical as well as cross-cultural psychology by highlighting the fundamental and universal importance of attachment and existential well-being for the mental health of young adults. Furthermore, it contributes to the research on the role of spirituality over the duration of adulthood [50, 54].

Especially existential well-being appears be relevant for the connection between attachment and mental health. By integrating findings on different components of existential well-being – e.g., with hope already predicting life-satisfaction in adolescents [55] and the presence of life meaning being connected to secure attachment [56] – future studies might also focus more closely on the relevance of these components in clinical settings. In addition, the inclusion of (positive) emotions might be prudent, as they also appear to have a strong connection to spirituality and mental health [57, 58]. Integrated with other relevant research in this area, our findings will hopefully inform future strategies for prevention and treatment of mood-related psychiatric burden in young adults with different cultural backgrounds.

## Data Availability

The datasets used and analysed during the current study are available from the corresponding author on reasonable request.

## Abbreviations

AV: Avoidant Attachment
AX: Anxious Attachment
BSI-18: Brief Symptom Inventory
ECR-RD: Experiences in Close Relationships - Revised
EWB: Existential Well-Being
GSI: Global Severity Index
MI-RSWB: Multidimensional Inventory of Religious/Spiritual Well-Being
RSWB: Religious-Spiritual Well-Being
RWB: Religious Well-Being
